# Tracking Motor Progression and Device‐Aided Therapy Eligibility in Parkinson's Disease

**DOI:** 10.1002/acn3.70188

**Published:** 2025-12-12

**Authors:** David Ledingham, Sahana Sathyanarayana, Charlotte B. Stewart, Robyn Iredale, Victoria Foster, Debra Galley, Mark Baker, Nicola Pavese

**Affiliations:** ^1^ Clinical Ageing Research Unit Newcastle University, Campus for Ageing and Vitality Newcastle upon Tyne UK; ^2^ Neurosciences Newcastle Upon Tyne NHS Foundation Trust Newcastle Upon Tyne UK; ^3^ Translational and Clinical Research Institute, the Medical School Newcastle University Newcastle Upon Tyne UK; ^4^ Department of Nuclear Medicine and PET Centre Aarhus University Hospital Aarhus Denmark

**Keywords:** device‐aided therapy, genetic subgroups, longitudinal analysis, motor complications, Parkinson's disease

## Abstract

**Objective:**

To characterise the progression of motor symptoms and identify eligibility for device‐aided therapies in Parkinson's disease, using both the 5‐2‐1 criteria and a refined clinical definition, while examining differences across genetic subgroups.

**Methods:**

We analysed 1205 individuals with sporadic and genetic Parkinson's disease from the Parkinson's Progression Markers Initiative (mean follow‐up: 5.6 ± 4.3 years). Kaplan–Meier analysis estimated time to meeting the 5‐2‐1 criteria (five or more daily levodopa doses, two or more hours of OFF time, or one or more hours of troublesome dyskinesia) and a stricter definition of eligibility for device‐aided therapy based on disabling, medication‐refractory symptoms and/or tremor. In the sporadic Parkinson's disease subgroup (*n* = 943), we assessed therapy initiation and clinical suitability, including potential contraindications. Genetic subgroup analyses explored differences in progression, eligibility timing and treatment uptake.

**Results:**

Among individuals with sporadic Parkinson's disease, 257 (27.3%) met the 5‐2‐1 criteria, with 25%, 50% and 75% doing so by 5.3, 8.2 and 10.7 years, respectively. A total of 176 (18.6%) met stricter eligibility criteria, with 50% doing so by 12 years. Only 25% of those meeting the 5‐2‐1 criteria initiated device‐aided therapy within 6.8 years. Most had no contraindications. Deep brain stimulation was the most used therapy. GBA and SNCA carriers met criteria earlier. LRRK2 carriers were more likely to initiate therapy, while PRKN carriers were less likely to meet eligibility thresholds.

**Interpretation:**

Eligibility for device‐aided therapy is common but underutilised. These findings highlight missed opportunities and support earlier, genotype‐informed treatment planning in Parkinson's disease.

## 1 Introduction

Motor complications such as OFF periods and dyskinesia are common in Parkinson's disease (PD) and significantly impact quality of life. A large cohort study by Gandhi et al. [1] reported that by 8–10 years post diagnosis, OFF periods affect nearly 60% of people with PD (PwP), and dyskinesia affects over 20%, with functional impairment present in more than half of those with OFF periods and a quarter with dyskinesia. Management includes increasing levodopa frequency and adding oral adjuncts. When these fail, device‐aided therapies (DAT) including deep brain stimulation (DBS), continuous infusion therapies, including levodopa carbidopa intestinal gel (LCIG), levodopa‐entacapone‐carbidopa intestinal gel (LECIG), continuous subcutaneous apomorphine infusion (CSAI) and continuous subcutaneous infusion of foslevodopa/foscarbidopa (CSCI) may be considered [2].

Many PwP who develop significant motor complications, often referred to as advanced Parkinson's disease (APD), though definitions vary and may extend beyond motor symptoms, are not promptly identified as candidates for DAT [3, 4, 5, 6]. Screening tools have been developed to guide clinicians to determine when PwP may benefit from medication optimisation, DAT or specifically DBS [6]. The 5‐2‐1 criteria are widely used due to their simplicity. A PwP meets the criteria for APD if they meet one of the following: ≥ 5 daily levodopa doses, ≥ 2 h of OFF time, or ≥ 1 h of troublesome dyskinesia [3].

Cross‐sectional studies have evaluated the 5‐2‐1 criteria across populations. Santos‐García et al. [7] found 19.6% of PwP at their centre met at least one criterion; Aldred et al. [8] reported that over 98% of those on LCIG met one or more. Malaty et al. [9] compared the 5‐2‐1 screen with physician judgement in a cohort of 4714 PwP and found 33% met criteria, with a positive predictive value of 35.7%. Moes et al. [6] reported that the criteria were 94% sensitive and 73% specific for identifying DAT candidates.

A disabling tremor is also common in PwP. Pasquini et al. [10] reported that 96.2% of PwP in the Parkinsons Progression Markers Initiative (PPMI) cohort exhibited tremor at least once over 7 years, with 20%–38% of tremor being unresponsive to dopaminergic therapy, depending on whether the tremor is present at rest or action. DBS is an established treatment for disabling tremor. More recently, MRI‐guided focused ultrasound thalamotomy (MRgFUS‐Th) has been FDA‐approved for tremor in Parkinson's, though it is not yet universally adopted for this indication.

While cross‐sectional studies estimate DAT use, longitudinal data on when PwP meets APD or DAT criteria are limited.

This study used up to 14‐years of PPMI follow‐up data to:

1. Determine when PwP met 5‐2‐1 criteria during the natural course of the disease.
2. Estimate timing of DAT eligibility.
3. Assess whether clinical features at the point of eligibility can help guide the selection of appropriate DAT modalities.
4. Evaluate DAT utilisation among eligible individuals.
5. Explore differences in the development of APD, eligibility for, and use of DAT across genetic subgroups.

## 2 Methods

### 2.1 Study Population

The PPMI study, started in 2010, is a multi‐centre observational study aiming to identify PD biomarkers. It has tracked participants for 14 years and is registered with ClinicalTrials.gov (NCT01141023).

Participants included those with both sporadic and genetic forms of PD. For sporadic PD, inclusion criteria required participants to be aged 30 or older, have a clinical diagnosis of PD for 2 years or less and not be on PD medication or expected to need it within 6 months of baseline. Patients with LRRK2 or GBA genetic variants were included if they had a clinical diagnosis of PD for up to 5 years until 2020, after which the criteria were adjusted to 2 years or less at screening, and they could be on dopaminergic medications at enrolment. Participants with SNCA, PRKN or Pink1 variants were eligible regardless of diagnosis timing or PD medication status.

For detailed inclusion and exclusion criteria, including genetic status determination, refer to the current protocol available at ‘002_Protocol_AM4_v3.0_23July2024_Final.pdf’. All participating sites obtained ethical approval, and written informed consent was secured from all participants.

### 2.2 Demographics and Clinical Assessments

Demographic information was recorded at study enrolment. PwP were reviewed at 6‐month intervals at which point the Movement Disorder Society—Unified Parkinson's Disease Rating Scale (MDS‐UPDRS) was performed. The participants vital signs, Hoehn & Yahr (H&Y) status, investigators assessment of cognitive status: ‘Cogstate’ and current medications were recorded at each visit. A Montreal Cognitive Assessment (MoCA) was performed at yearly intervals. As part of the study, PwP also undergo genetic analysis. At each in‐person visit, an MDS UPDRS III motor score was performed in both the OFF and ON states.

### 2.3 Criteria Assignment

The criteria used to define 5‐2‐1, and DAT eligibility are summarised in Table 1. To allow for stabilisation of dopaminergic therapy and minimise confounding from early treatment adjustments, we began collecting data on motor complications 1 year after initiation of anti‐parkinsonian medication. This decision was informed by clinical experience, where early motor fluctuations often reflect under‐ or over‐dosing rather than stable treatment response.

**TABLE 1:** Criteria for 5‐2‐1, tremor and device‐aided therapy exclusion.

Criteria	Description
Clinical criteria	
5‐2‐1 Criteria	**5**: ≥ 5 oral doses of levodopa per day (immediate or modified release). Additional dose if > 1 PRN preparation per day. **2**: ≥ 2 h OFF time within 24 h. Irrespective of severity. **1**: ≥ 1 h of dyskinesias and MDS UPDRS Part IV 4.2 score ≥ 3. (dyskinesias impact on activities to the point that the patient usually does not perform activities or does not usually participate in some social activities during dyskinetic episodes) **5‐2‐1**: Met any of the above three criteria.
Functionally disabling, medication refractory 5‐2‐1 criteria (FDMR‐5‐2‐1)	**5**: Met criteria for 5 above. **2**: ≥ 2 h OFF time within 24 h and MDS Part IV 4.4 score ≥ 3 (Fluctuations impact on the performance of activities during OFF to the point that the patient usually does not perform some activities or participate in some social interactions that are performed during ON periods). OFF periods were considered medication refractory if they continued despite two previous or current trials of adjuncts considered clinically or likely efficacious for the management of OFF periods [1]. **1**: Met criteria for 1 above and trialled/currently prescribed amantadine or amantadine contraindicated. Contraindications to amantadine included history of gastric ulceration, severe renal disease, epilepsy, psychosis and history of cardiovascular disorders. **FDMR 5‐2‐1:** Met any of the above three criteria
Functionally disabling medication refractory tremor (FDMR Tremor)	**Functionally Disabling**: MDS UPDRS II 2.10 score ≥ 3 (shaking or tremor causes problems with many of my daily activities). **Medication Refractory**: MDS UPDRS limb score (3.15, 3.16, 3.17) ≥ 3 (≥ 3 cm in maximal amplitude) and does not improve to ≤ 2 with usual dose of levodopa.
Exclusion criteria for device‐aided therapies	
DBS unsuitable	> 75 years old, dementia (MoCA ≤ 25), Impaired balance (H&Y ON score ≥ 3), active psychosis (MDS UPDRS item 1.2 ≥ 3).
DBS borderline	Aged 70–75 years, troublesome hallucinations (MDS UPDRS item 1.2 = 2).
CSCI/LCIG/LECIG unsuitable	Moderate–severe dementia (MoCA ≤ 17), active psychosis (MDS UPDRS item 1.2 ≥ 3).
CSCI/LCIG/LECIG borderline	Mild dementia (MoCA 18–25).
CSAI unsuitable	Orthostatic hypotension (systolic drop of ≥ 20 mmHg or a diastolic drop of ≥ 10 mmHg), active psychosis (MDS UPDRS item 1.2 ≥ 3), moderate–severe dementia (MoCA ≤ 17).
CSAI borderline	Mild dementia (MoCA 18–25).

Abbreviations: CSAI, continuous subcutaneous apomorphine infusion; CSCI, continuous subcutaneous infusion of foslevodopa/foscarbidopa; DBS, deep brain stimulation; LCIG, Levodopa carbidopa intestinal gel; LECIG, Levodopa‐entacapone‐carbidopa intestinal gel.

#### 2.3.1 Assignment of Eligibility Criteria for DAT

There are currently no universally accepted criteria for DAT eligibility. Definitions of ‘optimal medical therapy’ and ‘disabling motor symptoms’ vary across clinical trials, guidelines and health systems. To address this variability, we reviewed multiple sources, including inclusion criteria from consensus agreements, randomised controlled trials, the 2022 EAN/MDS‐ES guidelines, and the NHS England Clinical Commissioning Policy, to construct a strict and reproducible definition of DAT eligibility [3, 11, 12, 13, 14, 15, 16, 17]. We aimed to align our criteria closely with the 5‐2‐1 framework while ensuring clinical relevance.

We defined DAT eligibility using the term *functionally disabling, medication‐refractory (FDMR)‐2‐1 and/or tremor*, to reflect a more clinically meaningful threshold than the 5‐2‐1 criteria alone.

We retained ≥ 5 daily levodopa doses as a marker of treatment complexity, representing the upper limit of what is typically considered acceptable oral therapy. This decision was supported by the PPMI protocol, which encouraged optimal medication regimens, suggesting that escalation to this frequency was intentional and clinically justified. In the sporadic PD subgroup, individuals meeting this threshold had typically trialled or were concurrently using a median of two adjunctive therapies for motor complications (IQR 1–3), and 73.2% continued to experience motor complications despite this regimen. These findings, along with existing literature on the burden of frequent dosing and its impact on adherence, support the use of this threshold as a proxy for functional and therapeutic burden [18, 19, 20, 21].

To confirm that symptoms were functionally disabling, we required a moderate or greater rating on MDS‐UPDRS Part IV, Item 4.2 for dyskinesias and Item 4.4 for motor fluctuations. Medication refractoriness was defined based on available evidence and clinical consensus. Although no head‐to‐head trials have compared adjunctive therapies for motor fluctuations, Fox and colleagues have summarised the evidence base for their use, and a recent meta‐analysis by Vibuthi et al. has compared their clinical effects [2, 22]. Based on this, medication‐refractory OFF periods were defined as ≥ 2 h of daily OFF time despite failure of two or more adjunct therapies recognised as efficacious. Medication‐refractory dyskinesia was defined as ≥ 1 h of troublesome dyskinesia despite treatment with amantadine or its contraindication.

A PwP was considered to have FDMR tremor if they rated it as functionally disabling (MDS‐UPDRS item 2.10 score of moderate or worse) and showed no meaningful response to levodopa, in line with EAN/MDS‐ES and NHS England criteria [16, 17].

These criteria were intentionally strict to ensure that included individuals clearly met clinical thresholds for DAT.

#### 2.3.2 Assignment of Exclusion Criteria for DAT

Suitability for DAT was assessed using exclusion criteria derived from published recommendations [3, 23] and our current clinical practice.

Evaluations considered age, cognitive status, orthostatic hypotension, balance impairment and the presence of hallucinations or psychosis. Cognitive function was assessed using the PPMI Cogstate report and graded by MoCA scores. All inclusion and exclusion criteria are summarised in Table 1.

Data for exclusion criteria were taken at the time FDMR 5‐2‐1 criteria were met, or from the earliest available time point thereafter. Individuals were considered potentially suitable for MRgFUS‐thalamotomy if they met all DBS criteria except age, as age is not a contraindication for MRgFUS.

### 2.4 Statistical Analysis

Kaplan–Meier (KM) survival analysis was used to estimate the probability of individuals meeting eligibility criteria over time from diagnosis. Survival probabilities and time points were calculated and visualised using RStudio. The Cox Proportional Hazards Model assessed hazard ratios and differences between sporadic and genetic subgroups. All KM analyses were based on time from diagnosis unless otherwise stated.

Descriptive and inferential statistics were conducted using IBM SPSS (Version 29). Binary logistic regression evaluated whether clinical variables, age, cognition, orthostatic hypotension, Hoehn & Yahr stage (ON) and hallucinations predicted DAT utilisation. One‐way ANOVA compared age at DAT eligibility across genetic subgroups, with independent samples *t*‐tests used for pairwise comparisons between LRRK2 and sporadic groups. Bonferroni correction was applied for multiple comparisons, and statistical significance was set at *p* < 0.05.

## 3 Results

### 3.1 Study Population

We identified 1291 PwP in the PPMI database. Of these, 86 were excluded for having met 5‐2‐1 or FDMR tremor criteria prior to enrolment, or for initiating before meeting these criteria. The final cohort included 1205 PwP: 943 with sporadic PD; 145 with LRRK2 PD; 83 with GBA PD, 6 with both GBA and LRRK2 variants; 18 with SNCA PD, 9 with PRKN PD; and 1 with PINK1 PD. Participant demographics are shown in Table 2.

**TABLE 2:** Distribution of baseline variables by genetic subgroup.

Baseline variable	Mean	Standard deviation	Range	Data available
Age at onset (years)				
Total	60.2	10.3	19–84	1192
Sporadic	61.0	9.6	25–84	935
LRRK2	59.6	10.1	19–83	143
GBA	57.8	11.3	26–81	80
Age at diagnosis (years)				
Total	61.9	9.9	24–85	1205
Sporadic	62.6	9.3	34–85	943
LRRK2	61.8	9.2	32–84	145
GBA	59.1	10.8	29–82	83
Age at enrolment (years)				
Total	63.1	9.7	29–85	1205
Sporadic	63.4	9.4	35–85	943
LRRK2	64.5	9.1	34–85	145
GBA	61.6	10.8	32–82	83
Educational years (years)				
Total	15.9	3.41	0–32	1201
Sporadic	16.0	3.1	5–32	939
LRRK2	15.3	4.5	0–26	145
GBA	16.1	3.8	5–24	83
Length of follow‐up (years from diagnosis to last visit)				
Total	5.6	4.3	0–24.5	1205
Sporadic	4.8	4.2	0–15.4	943
LRRK2	8.8	3.4	0.8–16	145
GBA	7.7	3.2	0.5–13.8	83

	Percentage	Data available
Gender (Male:Female)		
Total	62.4:37.6	1205
Sporadic	65.4:34.6	943
LRRK2	49.7:50.3	145
GBA	54.2:45.8	83
Handedness (Right:Left:Ambidextrous)		
Total	88.2:8.9:2.9	1205
Sporadic	88.2:8.9:2.9	943
LRRK2	89.7:6.2:4.1	145
GBA	83.1:14.5:2.4	83
Race (White:Black:Asian:Other including multiracial)		
Total	93.3:1.7:1.2:3.4	1199
Sporadic	93.3:2.0:1.4:3.3	941
LRRK2	93.8:0:0.7:5.5	145
GBA	96.4:1.2:0:2.4	83

The number of participants under follow‐up declined over time due to staggered enrolment across the 14‐year study period, rather than significant attrition. Readers are referred to the number‐at‐risk tables in the KM plots for detailed counts at each time point.

### 3.2 Motor Symptoms

Findings from Kaplan–Meier survival analysis revealed the following patterns in the prevalence and progression of motor symptoms in individuals with sporadic PD (Tables 3 and 4):

**TABLE 3:** Milestone Table based on Kaplan–Meier Analysis detailing years to reach Event Threshold from diagnosis for participants with Sporadic PD.

Event threshold (%)	10	20	25	30	40	50	60	70	75	80
Dyskinesia										
1 h of dyskinesia	3.8	5.2	5.5	6.2	7.2	8.3	9.4	10.6	11.4	12
1 h of FD dyskinesia	11.3	12.4	—	—	—	—	—	—	—	—
1 h of FDMR dyskinesia	12.3	—	—	—	—	—	—	—	—	—
OFF time										
2 h OFF	4.4	6.2	6.5	7.6	8.8	9.7	10.6	11.3	12	12.5
2 h OFF FD	7.7	9.7	10.5	11.3	12	12.8	—	—	—	—
2 h OFF FDMR	9.6	11.3	11.7	12.6	—	—	—	—	—	—
Tremor										
FD tremor	4.8	9.9	11.6	12.2	—	—	—	—	—	—
FDMR tremor	12.2	—	—	—	—	—	—	—	—	—
5‐2‐1 Criteria and suitability for DAT										
5 Levodopa preparations	4.6	7.3	8.1	8.4	9.8	11.9	13	—	—	—
5‐2‐1	3.2	4.6	5.3	6.1	7.3	8.2	9.2	10.3	10.7	11.3
FDMR 5‐2‐1	7.5	9.8	10.8	11.3	11.7	12.2	12.6	12.8	—	—
FDMR 5‐2‐1 AND/OR FDMR tremor	6.6	9.4	10.3	11.1	11.6	12	12.3	12.7	—	—
DAT										
5‐2‐1 to DAT	2.3	4.6	6.8	—	—	—	—	—	—	—
FDMR 5‐2‐1 AND/OR FDMR tremor to DAT	4.3	—	—	—	—	—	—	—	—	—

*Note:* For criteria definitions, please see Table 1; – infers this event threshold was not reached, or NR < 10 during follow‐up.

Abbreviations: FD, functionally disabling; MR, medication refractory.

**TABLE 4:** Time to 5‐2‐1 and other outcome scores from diagnosis.

Outcome	Population	Number (%)	Median time to outcome (years)	Inter‐quartile range (years)	Range (years)	Data available
5 levodopa preparations	Total	237 (19.7%)	6.2	55.8	1.2–15.2	1205
	Sporadic	133 (14.1%)	6.5	5.1	1.4–13	943
	LRRK2	57 (39.3%)	6.1	4.7	1.2–15.2	145
	GBA	37 (44.6%)	5.4	3.5	1.7–11.3	83
2 h OFF	Total	363 (30.1%)	7.1	5.1	1.3–14.1	1205
	Sporadic	218 (23.1%)	7.5	5.6	1.3–14.1	943
	LRRK2	82 (56.6%)	7.1	4.3	1.4–13.6	145
	GBA	47 (56.6%)	5.8	4.3	1.8–11.2	83
1 h of FD dyskinesia	Total	49 (4.1%)	9.2	4.4	2.9–13.7	1205
	Sporadic	32 (3.4%)	10.1	3.2	2.9–13.7	943
	LRRK2	9 (6.2%)	8.8	2.7	4.1–12	145
	GBA	5 (6%)	6.5	3.6	4.7–9.1	83
5‐2‐1	Total	432 (35.9%)	6.1	4.9	1.2–13.8	1205
	Sporadic	257 (27.3%)	4.2	3.3	1.3–13.8	943
	LRRK2	97 (66.9%)	6.3	4.3	1.2–13.6	145
	GBA	60 (72.3%)	5.4	4.3	1.8–11.3	83
2 h OFF (FDMR)	Total	105 (8.7%)	8.8	3.6	2.4–13.7	1205
	Sporadic	51 (5.4%)	9.7	2.9	3.6–13.7	943
	LRRK2	33 (22.8%)	8	3.5	4.1–13.6	145
	GBA	14 (16.9%)	6.7	2.8	4.7–11.2	83
1 h of FDMR dyskinesia	Total	28 (2.3%)	9.2	4.5	2.9–13.7	1205
	Sporadic	20 (2.1%)	10.5	2.9	2.9–13.7	943
	LRRK2	3 (2.1%)	7.8	2.9	5–11.5	145
	GBA	3 (3.6%)	6.5	1.8	4.7–8.9	83
FDMR 5‐2‐1	Total	286 (23.7%)	6.6	4.7	1.2–13.7	1205
	Sporadic	158 (16.8%)	7.2	5.5	1.4–13.7	943
	LRRK2	70 (48.3%)	6.6	4.7	1.2–13.6	145
	GBA	44 (53%)	5.8	3	1.7–11.3	83
FD tremor	Total	131 (10.9%)	4.3	5.4	0–13.2	1205
	Sporadic	84 (8.9%)	4.5	6	0–13	943
	LRRK2	24 (16.6%)	3.9	5	0–13.2	145
	GBA	19 (22.9%)	3.5	4.3	0–11	83
FDMR tremor	Total	43 (3.6%)	4.2	3.1	1.5–12.3	1205
	Sporadic	34 (3.6%)	4.2	3.3	1.5–12.3	943
	LRRK2	4 (2.8%)	4.7	3.5	3.4–7.8	145
	GBA	4 (4.8%)	4.4	3.6	2.7–6.9	83
FDMR 5‐2‐1 AND/OR FDMR tremor	Total	308 (25.6%)	6.1	4.7	1.2–13.7	1205
	Sporadic	176 (18.6%)	6.1	5.3	1.4–13.7	943
	LRRK2	72 (49.7%)	6.5	4.7	1.2–13.6	145
	GBA	46 (55.4%)	5.4	3	1.7–11.3	83

*Note:* For criteria definitions, please see Table 1.

Abbreviations: FD, functionally disabling; MR, medication refractory.

#### 3.2.1 OFF Time

Experiencing at least 2 h of OFF time was common, with 25%, 50% and 75% of individuals reaching this threshold at 6.5, 9.7 and 12 years, respectively. Two hours of FD OFF time was less frequent, occurring in 25% and 50% of individuals by 10.5 and 12.8 years. FDMR OFF time of the same duration was rarer still, with 25% of individuals affected by 11.6 years.

#### 3.2.2 Dyskinesia

At least 1 h of dyskinesia was experienced by 25%, 50% and 75% of PwP by 5.5, 8.3 and 11.4 years, respectively. Functionally disabling dyskinesia was relatively uncommon, affecting 20% of individuals by 12.4 years. FDMR dyskinesia was even less prevalent, with only 10% of PwP developing it by 12.3 years.

#### 3.2.3 Tremor

Tremor was highly prevalent, with 98.5% of individuals having it documented at least once during follow‐up, the majority presenting with tremor at study entry, precluding analysis of time to onset. FD tremor was reported by 25% of PwP by 11.6 years, while FDMR tremor was experienced by only 10% by 12.2 years.

### 3.3 5‐2‐1

**FIGURE 1:**
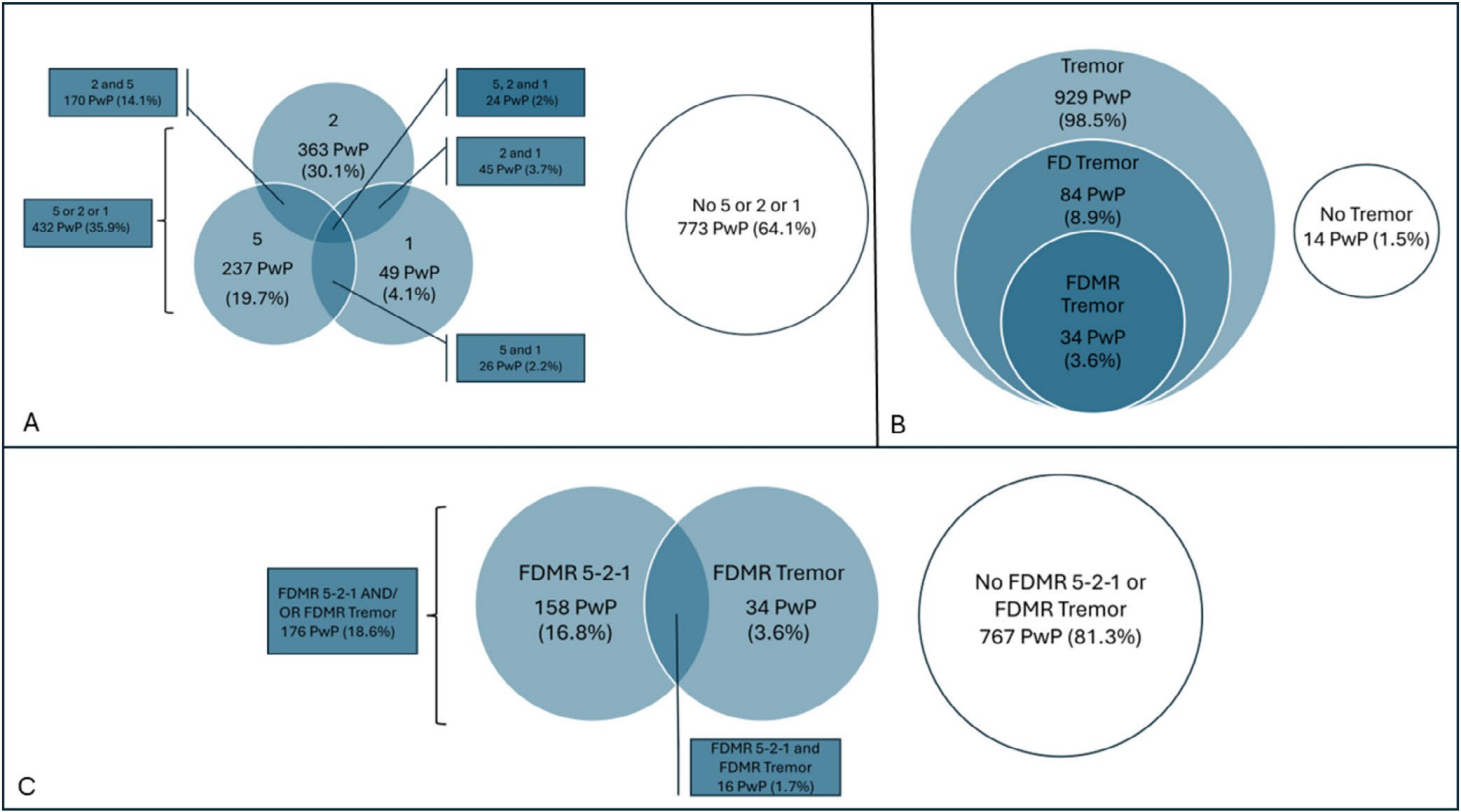
Proportion of PwP from the sporadic population (*n* = 943) who met 5‐2‐1 criteria and eligibility criteria for Device‐Aided Therapies at any point during follow‐up. (A) Number and Percentage of PwP meeting 5‐2‐1 criteria; 5 – 5 preparations of levodopa/24 h, 2 – 2 hours of OFF time (of any severity); 1 – 1 hour of troublesome dyskinesia. (B) Number and percentage of PwP who developed tremor, functionally disabling (FD) tremor and functionally disabling medication‐resistant tremor (FDMR) at any point during follow‐up. (C) Number and percentage of PwP who met FDMR 5‐2‐1 criteria and/or developed FDMR tremor at any point during follow‐up.

Out of 943 participants with sporadic PD, 257 (27.3%) met 5‐2‐1 criteria during follow‐up, with a median time from diagnosis of 4.2 years (IQR 3.3). Among the individual components, PwP were most likely to develop 2 h of OFF time (218 individuals, 23.1%) at a median of 7.5 years (IQR 5.6), followed by the need for five daily doses of levodopa (133 individuals, 14.1%) at a median of 6.5 years (IQR 5.1) and 1 h of troublesome dyskinesia (32 individuals, 3.4%) at a median of 10.1 years (IQR 3.2) (Figure 1A and Table 4).

**FIGURE 2:**
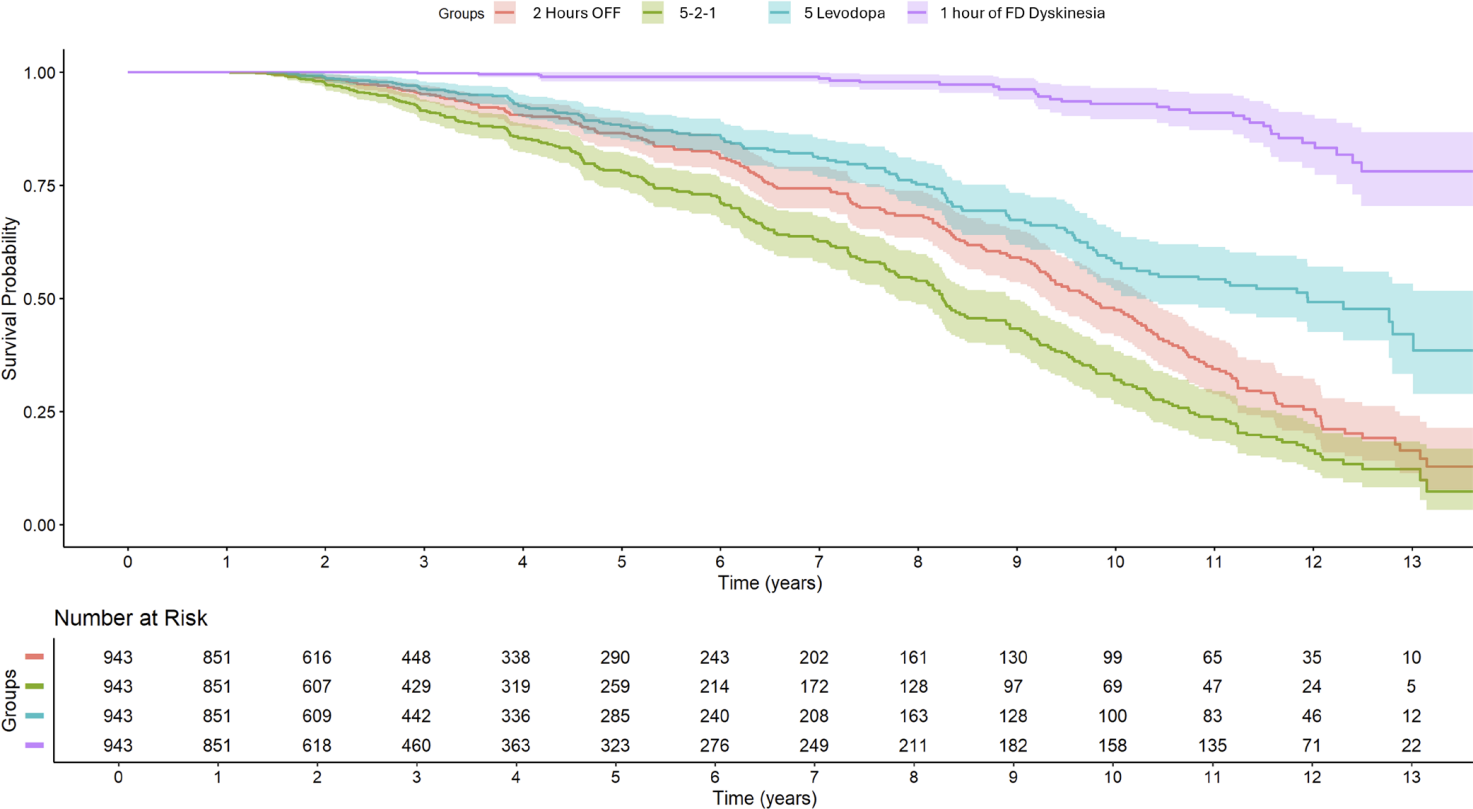
A Kaplan–Meier Survival curve for time from diagnosis to meeting 5‐2‐1 criteria for individuals with Sporadic PD*FD, functionally disabling/troublesome.

KM survival analysis demonstrated that 25%, 50%, and 75% of PwP met 5‐2‐1 criteria by 5.3, 8.2 and 10.7 years, respectively (Table 3 and Figure 2).

### 3.4 Suitability for DAT

Out of 943 participants with sporadic PD, 158 (16.8%) met FDMR 5‐2‐1 criteria during follow‐up, with a median time from diagnosis of 7.2 years (IQR 5.5). Among the individual components, PwP were most likely to require five doses of levodopa daily (14.1%), followed by FDMR 2‐h OFF time (5.4%, median 9.7 years, IQR 2.9), and FDMR dyskinesia (2.1%, median 7.8 years, IQR 2.9) (Figure 1C and Table 4).

Among the 51 PwP who developed FDMR 2‐h OFF time, the median number of adjunct therapies trialed or in use was three (range 2–6), with 60.8% having used three or more. MAO‐B inhibitors were the most used adjuncts (85.3%), followed by dopamine agonists (74.5%), COMT inhibitors (43.1%), extended‐release levodopa (51%) and istradefylline (3.9%). Of the 20 PwP who developed FDMR dyskinesia, 80% had used or were using amantadine, while 20% had one or more contraindications to its use (Tables S1 and S2).

KM analysis showed that 25% of PwP met FDMR 5‐2‐1 criteria by 10.8 years, and 50% by 12.2 years (Table 3). At the time of meeting these criteria, 67.4% were potentially suitable for DBS (50.3% suitable, 17.1% borderline), 78.4% for CSAI (76.7% suitable, 0.7% borderline) and 97.2% for levodopa infusion therapies (96.6% suitable, 0.6% borderline) (Tables 5 and 6).

**TABLE 5:** Percentage of participants with sporadic PD with contraindications to Device‐Aided Therapy at point of eligibility for Device‐Aided Therapy.

Potential contraindication to device‐aided therapy	Percentage	Data available
Age (years)		
> 75	23.3% (41)	176/176
70–75	23.9% (42)	176/176
< 70	52.8% (93)	176/176
Balance		
H&Y ON < 3	87.1% (148)	170/176
H&Y ON ≥ 3	12.9% (22)	170/176
Cognition		
No dementia	97% (160)	165/176
Mild dementia	0.6% (1)	165/176
Moderate–severe dementia	2.4% (4)	165/176
Orthostasis		
No orthostatic hypotension	78.2% (129)	165/176
Orthostatic hypotension	21.8% (36)	165/176
Psychosis		
No or non‐troublesome hallucinations	96.5% (166)	172/176
Troublesome hallucinations	2.3% (4)	172/176
Active psychosis	1.2% (2)	172/176

*Note:* For criteria definitions, please see Table 1.

Abbreviation: H&Y, Hoehn and Yahr score in the ON state.

**TABLE 6:** Suitability for device‐aided therapy by indication and therapy.

Device‐aided therapy	Suitable	Borderline	Unsuitable	Data available
FDMR 5‐2‐1				
DBS	50.3% (77)	17.1% (27)	32% (49)	153/158
CSAI	76.7% (115)	0.7% (1)	22.7% (34)	148/158
CSCI/LCIG/LECIG	96.6% (143)	0.6% (1)	2.7% (4)	150/158
FDMR tremor				
DBS	51.5% (17)	18.2% (6)	29.4% (10)	33/34
MRgFUSTh	85.3% (29)	—	12.1% (4)	22/34
FDMR 5‐2‐1 AND/OR FDMR tremor				
DBS	49.4% (84)	17.6% (30)	32.9% (56)	170/176

*Note:* For criteria definitions, please see Table 1.

Abbreviations: CSAI, continuous subcutaneous apomorphine infusion; CSCI, continuous subcutaneous infusion of foslevodopa/foscarbidopa; DBS, deep brain stimulation; FD, functionally disabling; LCIG, Levodopa carbidopa intestinal gel; LECIG, Levodopa‐entacapone‐carbidopa intestinal gel; MRgFUSTh, MRI guided focused ultrasound thalamotomy; MR, medication refractory.

A total of 83 PwP (8.9%) developed functionally disabling limb tremor during follow‐up (median 4.5 years, IQR 6.0). Of these, 34 (3.6% of the cohort) met criteria for FDMR tremor based on levodopa test scores (median 4.2 years, IQR 3.3). KM analysis showed that 10% of PwP developed FDMR tremor by 12.2 years (Table 3 and Figure 1B). At the time of meeting FDMR tremor criteria, 69.7% were potentially suitable for DBS (51.5% suitable, 18.2% borderline). An additional 12.1% were unsuitable for DBS due to age > 75 years. However, 85.3% were potentially suitable for MRgFUS‐thalamotomy, where age is not a contraindication (Tables 5 and 6).

In total, 176 PwP (18.6%) met criteria for FDMR 5‐2‐1 and/or FDMR tremor, with a median time of 6.1 years (IQR 5.3). KM analysis showed that 25% and 50% met one or both criteria by 10.3 and 12.0 years, respectively (Table 3 and Figure 1C). Among these, 67% were potentially suitable for DBS (49.4% suitable, 17.6% borderline) (Tables 5 and 6).

### 3.5 Proportion of PwP Who Underwent DAT by Eligibility Criteria

Of the 943 individuals with sporadic PD, 257 met 5‐2‐1 criteria. Among them, 32 (12.5%) underwent DAT during follow‐up, representing 3.4% of the total cohort. Of these, 31 (96.9%) received DBS and one (3.1%) received Duodopa. All underwent DAT after meeting 5‐2‐1 criteria; those who received DAT beforehand were excluded. KM analysis showed that 25% of those meeting 5‐2‐1 criteria were treated with DAT within 6.8 years of meeting criteria (See Table 3 and Figure S1).

Among the 176 PwP who met FDMR 5‐2‐1 and/or FDMR tremor criteria, 21 (11.9%) underwent DAT, all with DBS. KM analysis showed that 10% were treated within 4.3 years of meeting criteria (See Table 3 and Figure S2).

### 3.6 Differences Between Genetic Subgroups

Of the 1205 PwP included, 262 carried genetic variants: 145 had LRRK2 PD, 83 had GBA PD, and a further 36 had rarer genetic variants. Among these, 66.9% of those with LRRK2 PD and 72.3% with GBA PD met 5‐2‐1 criteria, at a median of 6.3 ± 4.3 and 5.4 ± 4.3 years, respectively (Table 4). The number of individuals meeting 5‐2‐1, FDMR 5‐2‐1, and tremor criteria across the full cohort (including both genetic and sporadic PD) is shown in Figure S3.

#### 3.6.1 Time to 5‐2‐1 Criteria

A Cox proportional hazards model assessed the likelihood of meeting 5‐2‐1 criteria across genetic subgroups, using sporadic PD as the reference. GBA carriers had a significantly higher hazard (HR = 1.86, 95% CI: 1.40–2.48, *p* < 0.001), as did SNCA carriers (HR = 2.21, 95% CI: 1.24–3.96, *p* = 0.007). PRKN carriers had a significantly lower hazard (HR = 0.13, 95% CI: 0.02–0.96, *p* = 0.046). No significant differences were observed for LRRK2, LRRK2 + GBA, or PINK1. The model was statistically significant (Likelihood ratio, Wald, and Score tests all *p* < 0.0001; Figure S4).

#### 3.6.2 Time to DAT Eligibility (FDMR 5‐2‐1 and/or FDMR Tremor)

A separate Cox model showed that GBA carriers met DAT eligibility criteria earlier during follow‐up (HR = 2.39, *p* < 0.001). No other subgroups showed significant differences. The model had a concordance of 0.589 and was statistically significant (*p* < 0.01; Figure S5).

#### 3.6.3 DAT After Meeting 5‐2‐1 Criteria

GBA (HR = 1.94, *p* = 0.045), LRRK2 (HR = 2.36, *p* = 0.001) and LRRK2 + GBA (HR = 5.55, *p* = 0.005) carriers who met 5‐2‐1 criteria were more likely to initiate DAT. No significant differences were observed for PRKN or SNCA. Model concordance was 0.616 (*p* < 0.01; Figure S6).

#### 3.6.4 DAT After Meeting FDMR 5‐2‐1 and/or FDMR Tremor Criteria

Similarly, among those eligible, GBA (HR = 2.16, *p* = 0.039), LRRK2 (HR = 3.00, *p* < 0.001) and LRRK2 + GBA (HR = 7.17, *p* = 0.001) carriers initiated DAT earlier or more frequently. No significant differences were found for PRKN or SNCA. Model concordance was 0.655 (*p* < 0.01; Figure S7).

#### 3.6.5 Exploring the Role of Clinical Characteristics in the Use of DAT

To investigate whether the increase in utilisation of DAT in the LRRK2 group was driven by clinical characteristics, a binary logistic regression was conducted. The model was statistically significant (*χ* ^2^(11) = 35.42, *p* < 0.001), explaining 19.2% of the variance in DAT utilisation. Age was the only significant predictor: Younger individuals were more likely to undergo DAT (OR = 0.923, *p* < 0.001). Other variables, including cognition, orthostatic hypotension, H&Y (ON) stage, and hallucinations, were not significant.

A one‐way ANOVA confirmed significant differences in age across genetic subgroups (*F*(5, 302) = 7.13, *p* < 0.001), with a moderate effect size (*η* ^2^ = 0.106). An independent samples t‐test showed that the LRRK2 group was younger than the sporadic group at the time of DAT eligibility (*M* = 65.17 vs. 67.63 years), though this difference did not reach statistical significance after correction for multiple comparisons (*p* = 0.065, Cohen's *d* = 0.26).

## 4 Discussion

This is the first study to evaluate the development of APD (as defined by meeting 5‐2‐1 screening criteria) longitudinally, and eligibility for DAT in a large cohort of people with early PD. The findings have important implications for clinical practice and healthcare resource planning. Individuals meeting 5‐2‐1 criteria have been shown to experience higher rates of falls, hospitalisations, reduced quality of life, and greater dissatisfaction with treatment compared to those who do not meet these criteria [9]. While our cohort is drawn from a highly motivated research population with good access to care, our findings are consistent with cross‐sectional studies reporting that 20%–33% of individuals meet 5‐2‐1 criteria, yet only a minority receive DAT [7, 9]. In the OBSERVE‐PD study, Fasano et al. [24] found that 51% of patients were classified as having APD, yet only 28.6% were receiving DAT. Similarly, the PARADISE study reported an APD prevalence of 38.2%, with just 5.7% of patients receiving DAT [25]. However, these prior studies do not capture the timing of eligibility or treatment uptake. By contrast, our longitudinal approach provides novel insights into the natural history of motor complications and the evolving window of DAT eligibility, which may help refine screening strategies and support earlier intervention in routine clinical settings.

Using strict, clinically relevant criteria for medication‐refractory symptoms, we identified when individuals met thresholds for DAT consideration and assessed subsequent treatment uptake. Despite a substantial proportion of PwP meeting eligibility criteria, use of DAT remained low, highlighting a persistent gap between clinical need and treatment delivery. Interestingly, time to DAT initiation was longer in the subgroup meeting FDMR 5‐2‐1AND/OR Tremor criteria compared to those meeting 5‐2‐1 criteria alone. This may reflect the smaller sample size and shorter follow‐up in the FDMR group but could also suggest that individuals who meet stricter eligibility thresholds later in the disease course may be more hesitant to pursue DAT or may be perceived as less suitable despite meeting clinical criteria. Further investigation into patient and clinician decision‐making in this group has been previously recognised as highly important [26].

The 5‐2‐1 criteria, while sensitive, may overestimate DAT eligibility if used in isolation. To enhance its specificity, particularly in capturing functionally disabling, medication‐refractory symptoms including tremor, we propose a refined framework: **FDMR 5‐2‐1 and/or TREMOR**. This approach retains the simplicity of the original criteria while incorporating clinically meaningful thresholds for tremor‐related disability and treatment resistance. While various studies have applied different criteria to identify candidates for DAT, our approach aimed to reflect a more pragmatic and clinically relevant threshold by incorporating functional disability and medication refractoriness. While the FDMR framework was designed to aid clinicians in identifying individuals who may meet clinical thresholds for DAT consideration, we fully acknowledge that patient preferences are central to treatment decisions. The framework is not intended to replace shared decision‐making, but rather to support it by providing a reproducible and clinically grounded reference point for when DAT might be considered.

We retained a threshold of ≥ 5 daily doses of levodopa as a marker of treatment complexity, recognising it as the upper limit of what is typically considered acceptable oral therapy. This threshold also reflects a substantial treatment burden, which is often overlooked. Frequent dosing requires strict adherence to timing, often avoiding food intake around medication, and can disrupt daily routines. The need to carry medication, manage complex schedules, and cope with the consequences of missed doses may negatively impact autonomy, adherence, and quality of life [18, 19, 21]. Despite its relevance, the burden of frequent levodopa dosing has not been systematically studied in PD [18]. We support the inclusion of treatment complexity in DAT eligibility assessments and highlight the need for further research into how medication regimens affect patient well‐being and decision‐making in Parkinson's disease.

Interestingly, although many PwP reported functionally disabling tremor, only a minority met criteria for FDMR tremor. This suggests that *medication‐responsive* tremor is often a prominent feature of OFF periods rather than a distinct, medication‐refractory symptom. This distinction is clinically relevant, particularly for DBS targeting, where FDMR tremor may require specific electrode placement (e.g., zona incerta or VIM). Careful levodopa responsiveness testing remains essential in differentiating tremor subtypes prior to surgery.

Our group previously observed that PwP with certain genetic variants, particularly LRRK2, were more likely to undergo DAT than those with sporadic PD (Ledingham et al., under submission). The current findings support and extend this: individuals with LRRK2 PD met DAT eligibility at similar rates to those with sporadic PD but were significantly more likely to proceed to treatment. While younger age may partly explain this, our analysis suggests genetic factors may independently influence treatment decisions. This does not imply faster disease progression in LRRK2 PD, which is inconsistent with current understanding, but may reflect differences in clinical confidence, patient preferences, or perceived predictability of disease course.

In contrast, individuals with GBA or SNCA variants developed motor complications earlier, consistent with prior studies [27, 28, 29, 30, 31, 32]. Consistent with this finding, those with GBA mutations, particularly individuals carrying both GBA and LRRK2 variants, were more likely to initiate DAT than those with LRRK2 mutations or sporadic PD alone. Reflecting this, those with GBA mutations, particularly individuals with both GBA and LRRK2 variants, were more likely to initiate DAT than those with LRRK2 mutations or sporadic PD alone. Interestingly, while our data suggest that individuals with both GBA and LRRK2 mutations progressed more rapidly than those with only one variant, previous studies have reported a slower rate of decline in this group. This discrepancy may be due to the small number of such cases in our cohort or differences in the specific mutations involved, as both GBA and LRRK2 variants can vary in severity [33, 34]. Conversely, individuals with PRKN‐associated PD were less likely to meet DAT criteria, consistent with their typically milder phenotype [28, 31]. These findings underscore the importance of genotype‐informed care pathways and support further research into personalised treatment strategies.

Several limitations should be acknowledged. This study was a retrospective analysis of a well‐characterised, research‐enriched population of PwP, with predominantly tremor‐dominant presentations, relatively high baseline functioning, minimal cognitive impairment, and good access to care [35]. These factors may limit generalisability to broader clinical populations.

Geographical differences in medication availability and healthcare systems may affect the applicability of DAT eligibility criteria. For example, Rytary is commonly used in North America and may reduce the frequency of daily dosing, potentially affecting the ‘5’ component of the 5‐2‐1 criteria. However, our refined FDMR 5‐2‐1 framework accounted for this by treating Rytary as an extended‐release preparation equivalent to an adjunct. Individuals on Rytary who continued to experience disabling motor complications would still be captured by the other components of the framework. Additionally, because our cohort is drawn from a study initiated in 2010, individuals who met DAT criteria in more recent years may have received therapies such as continuous subcutaneous apomorphine or foslevodopa infusion, which were not widely available earlier in the study period. This temporal variation in therapy availability, particularly the recent introduction of CSCI globally and CSAI in the United States, should be considered when interpreting DAT utilisation.

The dataset lacks information on patient preferences, insurance status, and whether individuals were offered but declined DAT. Some exclusion criteria relevant to DAT, such as mood disorders, impulse control issues and freezing, were not systematically captured. Cognitive impairment was assessed using Cogstate and MoCA, which may not reflect all domains relevant to DBS candidacy, where comprehensive neuropsychological evaluation is typically recommended. These factors may contribute to the observed gap between clinical eligibility and treatment uptake.

Certain genetic subgroups, such as PRKN and SNCA, were small and may be underpowered to detect statistically significant differences. Ideally, these findings should be interpreted with caution and validated in larger cohorts if possible.

Additionally, the in‐clinic levodopa challenge used to assess tremor responsiveness relied on participants withholding their usual evening dose and taking it the following morning. This approach assumes accurate dosing and may not reliably distinguish medication‐refractory tremor, especially given evidence suggesting the coexistence of dopamine‐responsive and dopamine‐resistant tremor phenotypes. In some cases, an insufficient levodopa dose may have led to misclassification.

As an observational study, PPMI is subject to attrition over time. Participant dropouts may introduce bias, particularly if related to disease progression or treatment decisions. Kaplan–Meier survival analysis, while useful, has inherent limitations in this context.

We also need to acknowledge that, while still a subject of debate, earlier initiation of DAT may offer significant benefits, including improved motor symptom control, reduced treatment burden, and enhanced quality of life [36, 37, 38]. Although our study applied strict criteria to define medication‐refractory (FDMR) states, we acknowledge that these thresholds may be too conservative to capture all individuals who could benefit from earlier intervention.

Despite the discussed limitations, this study represents the first longitudinal evaluation of DAT eligibility using the 5‐2‐1 framework. By integrating genetic subgroups and proposing a refined clinical threshold, we provide a comprehensive and pragmatic analysis of motor progression and treatment gaps in PD. Future work should focus on developing predictive models to identify individuals likely to progress to FDMR status, enabling proactive treatment planning and timely referral for DAT. In parallel, efforts should explore barriers to the use of DAT among eligible individuals and validate refined eligibility criteria in real‐world clinical settings. Additionally, evaluating outcomes following DAT in different genetic subgroups would be a valuable area for future study.

## Author Contributions

D.L., M.B. and N.P. conceptualised the investigation and developed the methodology. D.L. performed formal analysis of the data. S.S., R.I. and C.B.S. validated the analysis. D.L., C.B.S., S.S., V.F., R.I., D.G. and N.P. contributed to conducting the investigation and collecting data. D.L. and N.P. prepared the original draft document. All authors contributed to the review of draft documents. All authors read and approved the final manuscript.

## Conflicts of Interest

The authors declare no conflicts of interest.

## Supporting information


**Figure S1:** A Kaplan–Meier Survival curve for time from meeting 5‐2‐1 criteria to DAT for individuals with Sporadic PD.

**Figure S2:** A Kaplan–Meier Survival curve for time from meeting FDMR 5‐2‐1 AND/OR FDMR Tremor criteria to DAT for individuals with Sporadic PD.

**Figure S3:** Proportion of PwP from the total population (*n* = 1205, including genetic and sporadic subgroups) who met 5‐2‐1 criteria and eligibility criteria for Device‐Aided Therapies at any point during follow‐up. (A) Number and Percentage of PwP meeting 5‐2‐1 criteria; 5 – 5 preparations of levodopa/24 h, 2 – 2 hours of OFF time (of any severity); 1 – 1 hour of troublesome dyskinesia. (B) Number and percentage of PwP who developed tremor, functionally disabling (FD) tremor and functionally disabling medication resistant tremor (FDMR) at any point during follow‐up. (C) Number and percentage of PwP who met FDMR 5‐2‐1 criteria and/or developed FDMR tremor at any point during follow‐up.

**Figure S4:** A Kaplan–Meier Survival Curve and Cox Proportional Hazards Model for time from diagnosis to meeting 5‐2‐1 criteria for genetic subgroups of PD. *Bold text indicates statistical significance. The sporadic PD subgroup was used as the reference category for the Cox Proportional Hazards Model.

**Figure S5:** A Kaplan–Meier Survival Curve and Cox Proportional Hazards Model for time from diagnosis to meeting FDMR 5‐2‐1 AND/OR FDMR Tremor criteria for genetic subgroups of PD. *Bold text indicates statistical significance. The sporadic PD subgroup was used as the reference category for the Cox Proportional Hazards Model.

**Figure S6:** A Kaplan–Meier Survival Curve and Cox Proportional Hazards Model for time from 5‐2‐1 criteria and starting DAT for genetic subgroups of PD. *Bold text indicates statistical significance. The sporadic PD subgroup was used as the reference category for the Cox Proportional Hazards Model.

**Figure S7:** A Kaplan–Meier Survival Curve and Cox Proportional Hazards Model for time from meeting FDMR 5‐2‐1 AND/OR FDMR Tremor criteria and starting DAT for genetic subgroups of PD. *Bold text indicates statistical significance. The sporadic PD subgroup was used as the reference category for the Cox Proportional Hazards Model.

**Table S1:** Number of adjuncts trialled by individuals with Sporadic PD prior to meeting functionally disabling medication refractory 2 h OFF criteria.
**Table S2:** Adjuncts trialled by individuals with sporadic PD prior to reaching criteria for functionally disabling, medication refractory 2 h OFF and 1 h of Dyskinesia.

## Data Availability

The datasets generated and/or analysed during the current study are freely available following written request from https://www.ppmi‐info.org/access‐data‐specimens/download‐data.
