# Supplementary figures and images for: Tracking Motor Progression and Device‐Aided Therapy Eligibility in Parkinson's Disease
Source: Ann Clin Transl Neurol. 2025 Dec 12;13(5):958–71. doi: 10.1002/acn3.70188 (PMC13161881; doi:10.1002/acn3.70188)

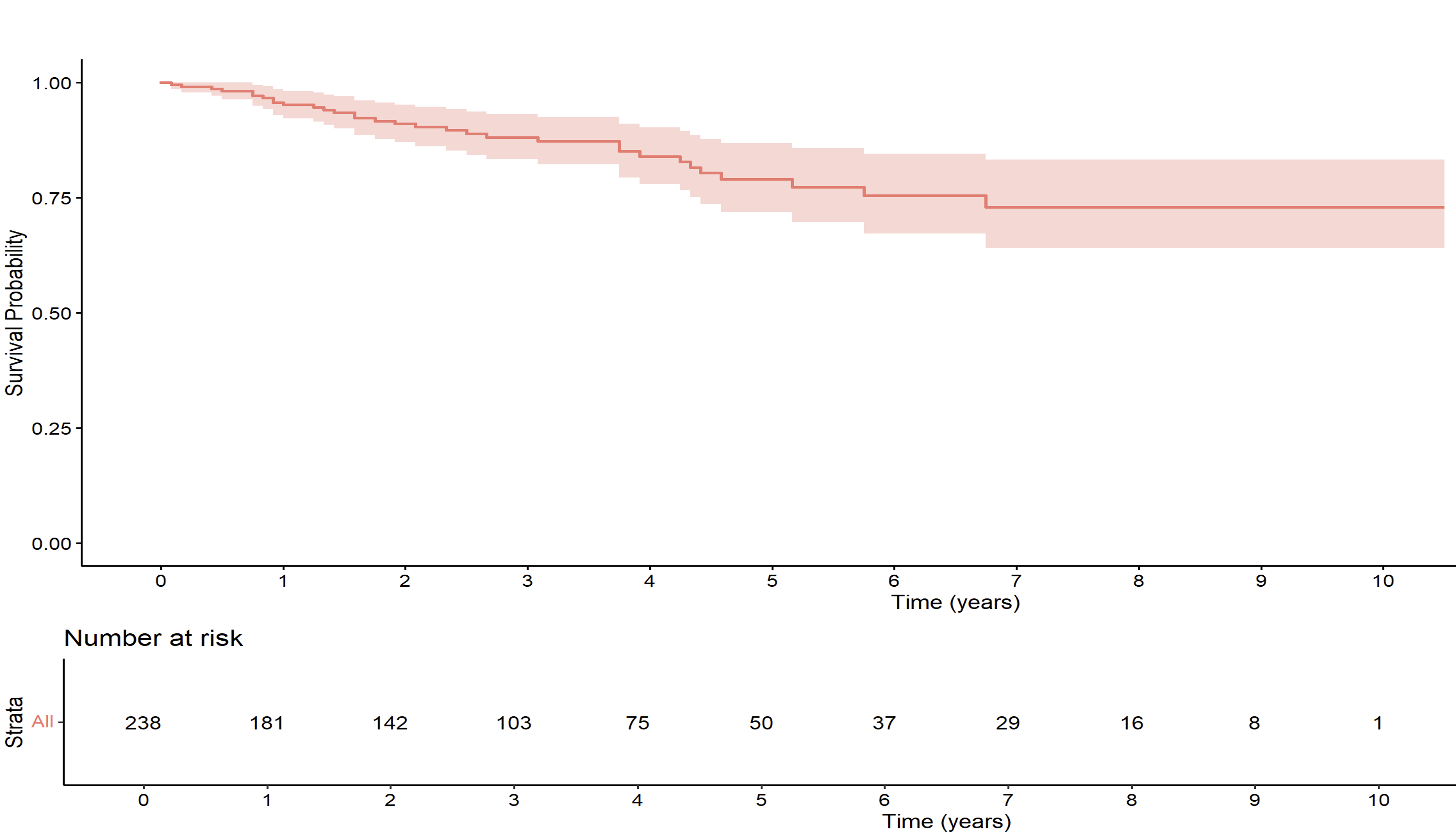

Supplement: Supplementary file 1 — Figure S1: A Kaplan–Meier Survival curve for time from meeting 5‐2‐1 criteria to DAT for individuals with Sporadic PD. [file ACN3-13-958-s001.tif]

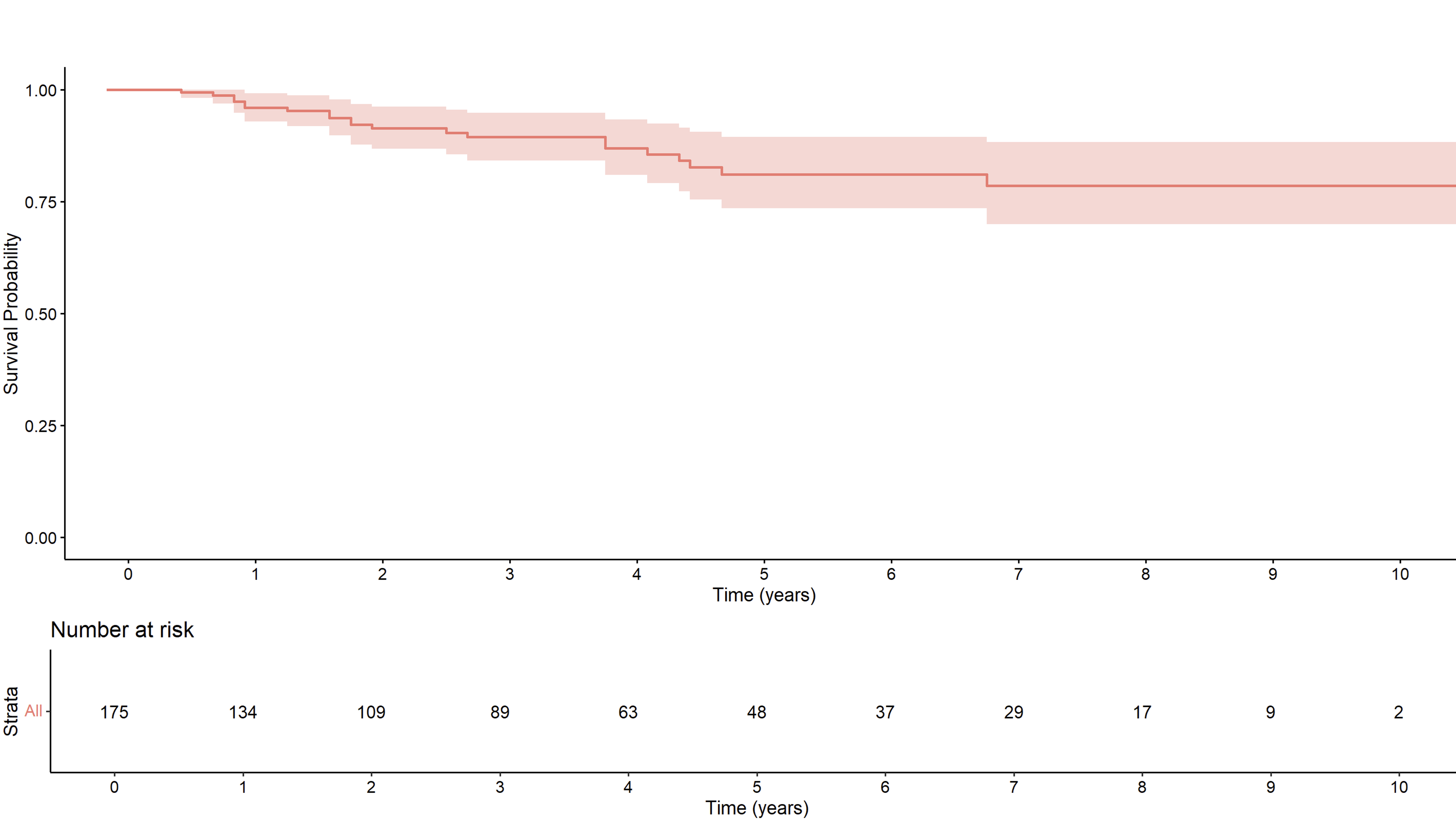

Supplement: Supplementary file 2 — Figure S2: A Kaplan–Meier Survival curve for time from meeting FDMR 5‐2‐1 AND/OR FDMR Tremor criteria to DAT for individuals with Sporadic PD. [file ACN3-13-958-s002.tif]

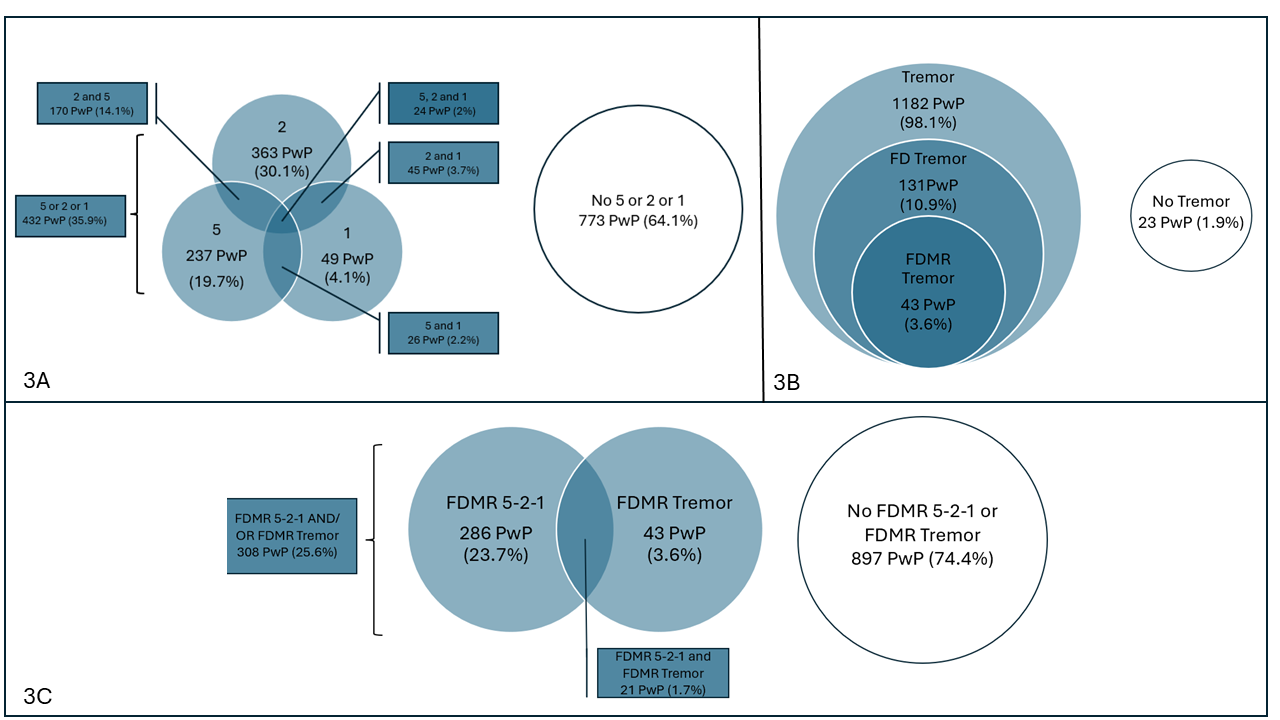

Supplement: Supplementary file 3 — Figure S3: Proportion of PwP from the total population (n = 1205, including genetic and sporadic subgroups) who met 5‐2‐1 criteria and eligibility criteria for Device‐Aided Therapies at any point during follow‐up. (A) Number and Percentage of PwP meeting 5‐2‐1 criteria; 5 – 5 preparations of levodopa/24 h, 2 – 2 hours of OFF time (of any severity); 1 – 1 hour of troublesome dyskinesia. (B) Number and percentage of PwP who developed tremor, functionally disabling (FD) tremor and functionally disabling medication resistant tremor (FDMR) at any point during follow‐up. (C) Number and percentage of PwP who met FDMR 5‐2‐1 criteria and/or developed FDMR tremor at any point during follow‐up. [file ACN3-13-958-s004.tif]

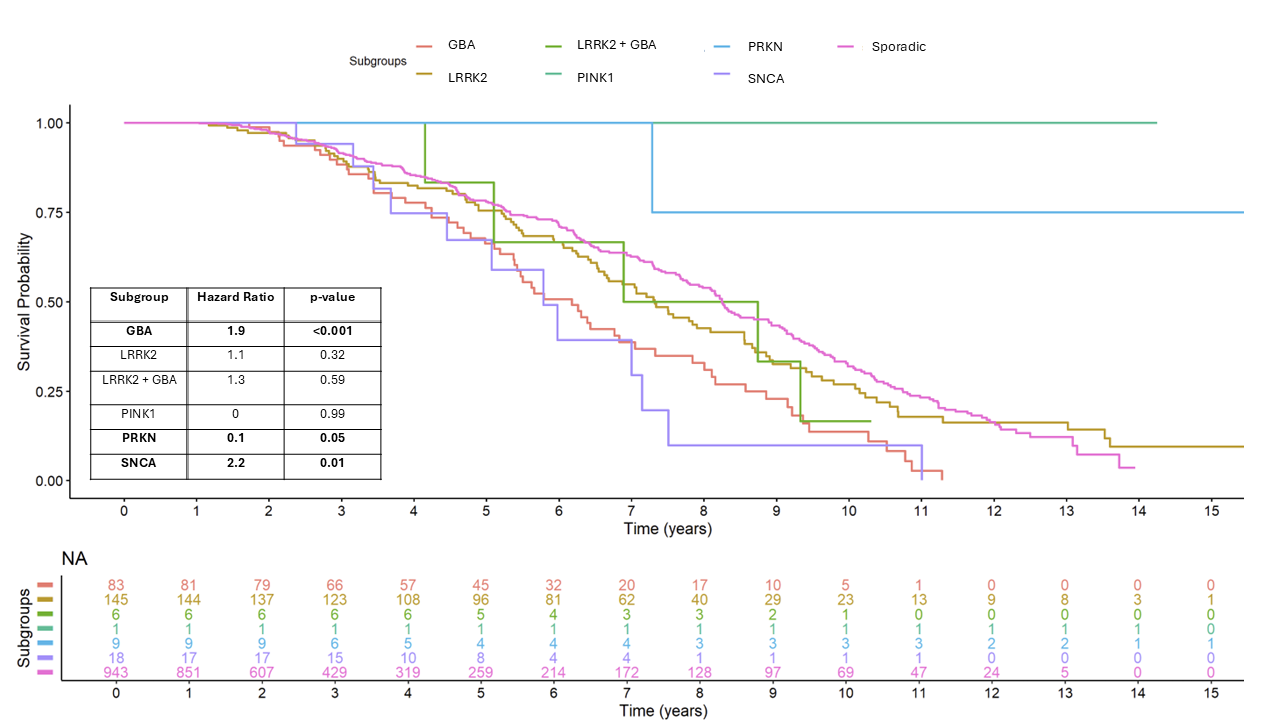

Supplement: Supplementary file 4 — Figure S4: A Kaplan–Meier Survival Curve and Cox Proportional Hazards Model for time from diagnosis to meeting 5‐2‐1 criteria for genetic subgroups of PD. *Bold text indicates statistical significance. The sporadic PD subgroup was used as the reference category for the Cox Proportional Hazards Model. [file ACN3-13-958-s003.tif]

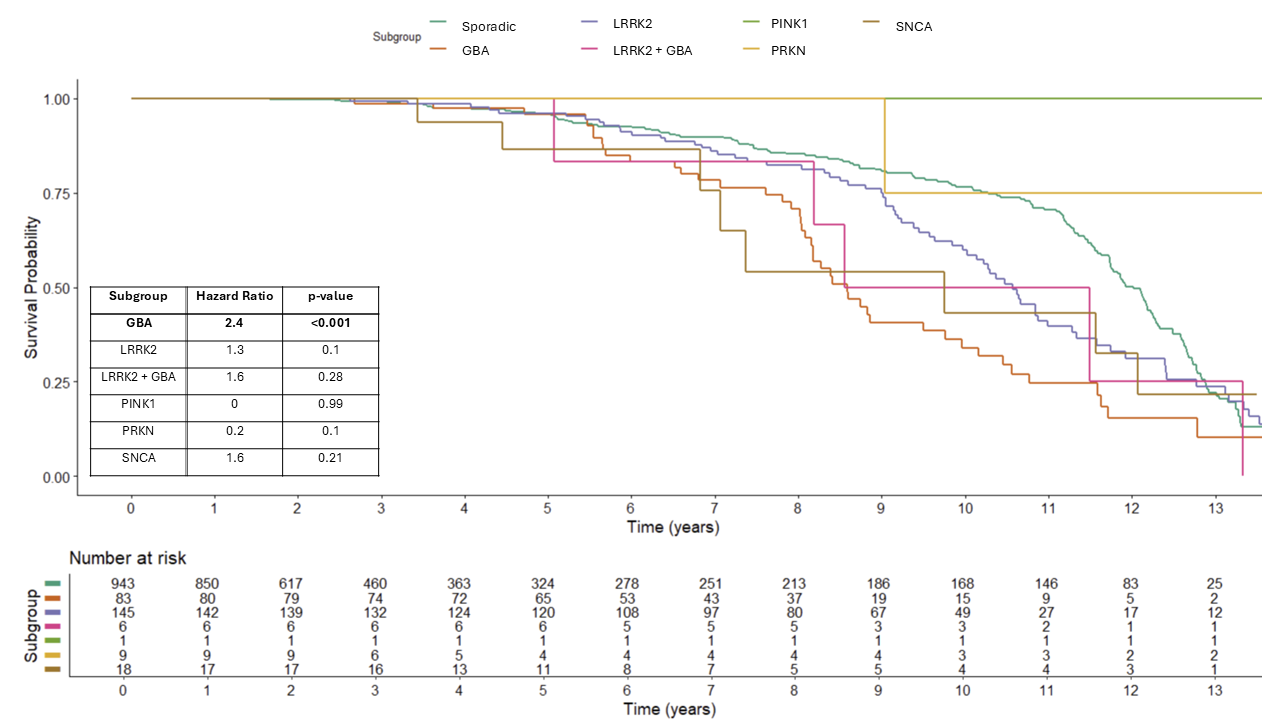

Supplement: Supplementary file 5 — Figure S5: A Kaplan–Meier Survival Curve and Cox Proportional Hazards Model for time from diagnosis to meeting FDMR 5‐2‐1 AND/OR FDMR Tremor criteria for genetic subgroups of PD. *Bold text indicates statistical significance. The sporadic PD subgroup was used as the reference category for the Cox Proportional Hazards Model. [file ACN3-13-958-s006.tif]

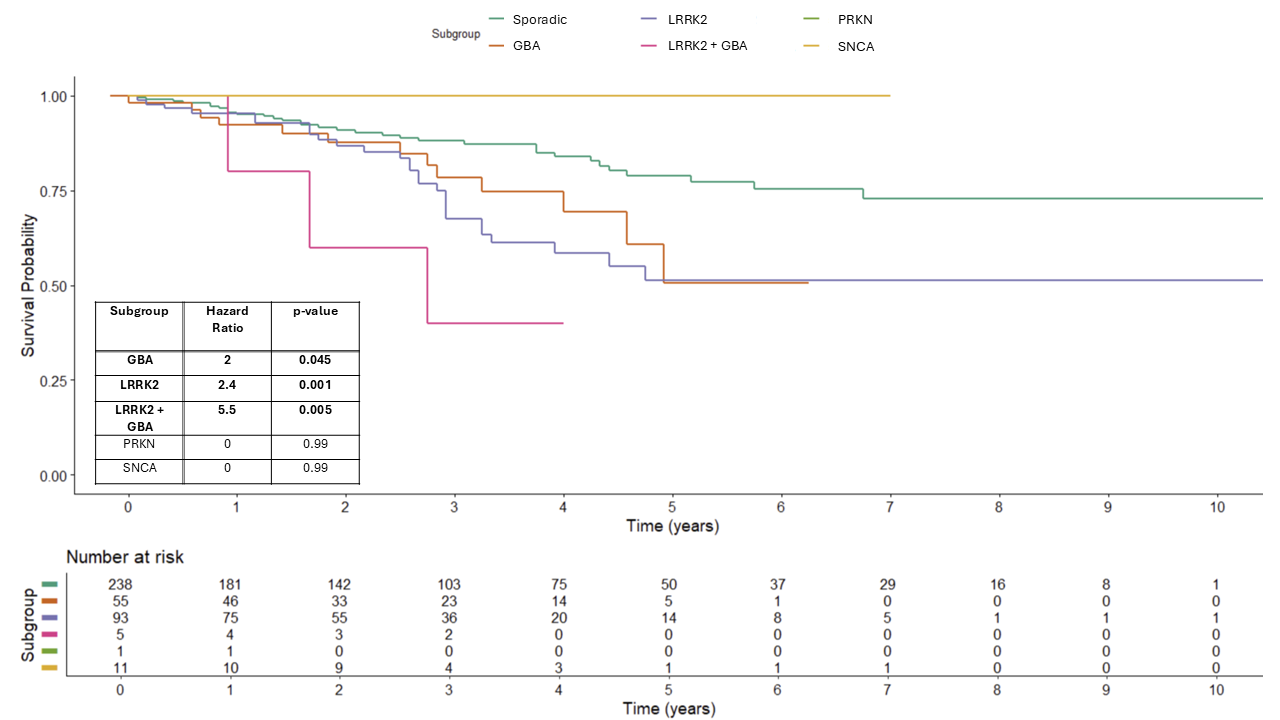

Supplement: Supplementary file 6 — Figure S6: A Kaplan–Meier Survival Curve and Cox Proportional Hazards Model for time from 5‐2‐1 criteria and starting DAT for genetic subgroups of PD. *Bold text indicates statistical significance. The sporadic PD subgroup was used as the reference category for the Cox Proportional Hazards Model. [file ACN3-13-958-s005.tif]

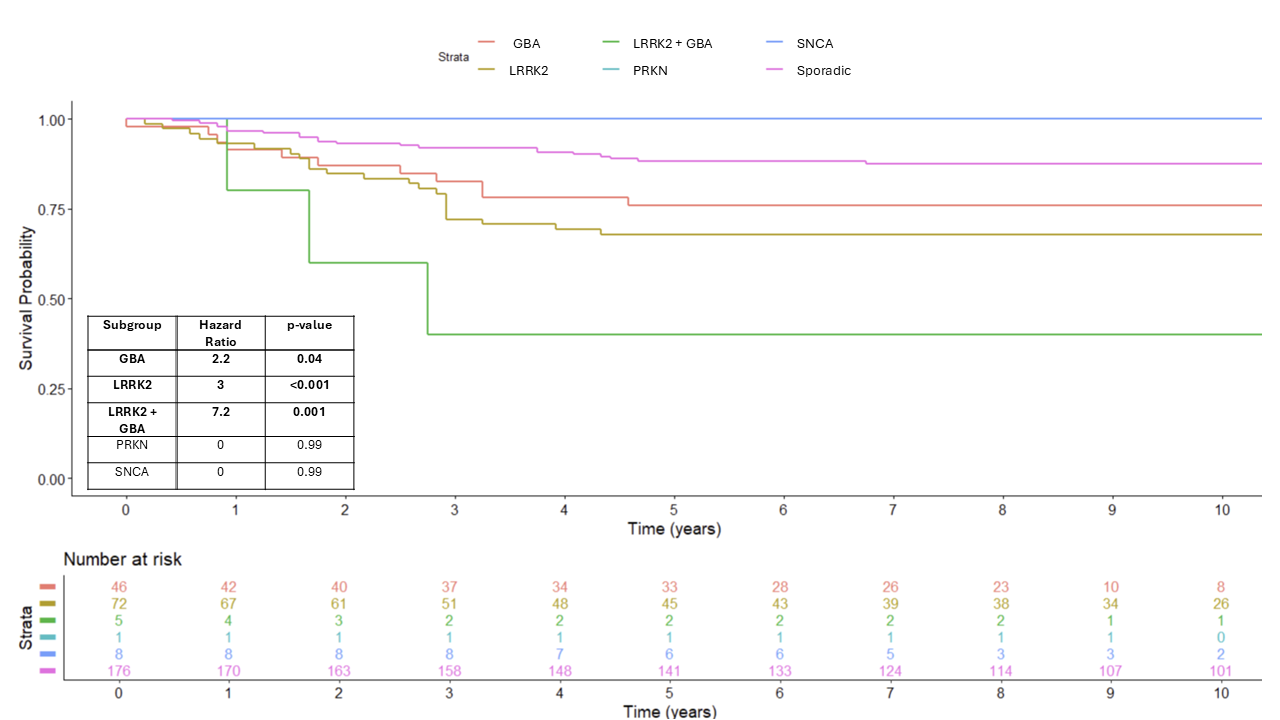

Supplement: Supplementary file 7 — Figure S7: A Kaplan–Meier Survival Curve and Cox Proportional Hazards Model for time from meeting FDMR 5‐2‐1 AND/OR FDMR Tremor criteria and starting DAT for genetic subgroups of PD. *Bold text indicates statistical significance. The sporadic PD subgroup was used as the reference category for the Cox Proportional Hazards Model. [file ACN3-13-958-s008.tif]
