# Supplementary material for: Tracking Motor Progression and Device‐Aided Therapy Eligibility in Parkinson's Disease
Source: Ann Clin Transl Neurol. 2025 Dec 12;13(5):958–71. doi: 10.1002/acn3.70188 (PMC13161881; doi:10.1002/acn3.70188)
Supplement: Supplementary file 8 — Table S1: Number of adjuncts trialled by individuals with Sporadic PD prior to meeting functionally disabling medication refractory 2 h OFF criteria. Table S2: Adjuncts trialled by individuals with sporadic PD prior to reaching criteria for functionally disabling, medication refractory 2 h OFF and 1 h of Dyskinesia. [file ACN3-13-958-s007.docx]

Supplementary Table 1: Number of adjuncts trialled by individuals with Sporadic PD prior to meeting functionally disabling medication refractory 2 hours OFF criteria

| **Number of adjuncts trialled** | **Percentage of PwP** | **Cumulative percentage** | **Data available** |
| --- | --- | --- | --- |
| **2** | 39.2% (20) | 100% (51) | 51/51 |
| **3** | 39.2% (20) | 60.8% (31) | 51/51 |
| **4** | 11.8% (6) | 21.6% (11) | 51/51 |
| **5** | 7.8% (4) | 9.8% (5) | 51/51 |
| **6** | 2% (1) | 2% (1) | 51/51 |

*PD – Parkinson’s Disease, PwP – People with Parkinson’s. For criteria definitions, please see Table 1

Supplementary Table 2: Adjuncts trialled by individuals with sporadic PD prior to reaching criteria for functionally disabling, medication refractory 2 hours OFF and 1 hour of Dyskinesia

| **Treatment trialled or Contraindication** | | **Percentage trialled** | **Data Available** |
| --- | --- | --- | --- |
| **2 hours OFF (FDMR)** | *COMTi* | 43.1% (22) | 51/51 |
|  | *MAO-Bi* | 86.3% (44) | 51/51 |
|  | *Dopamine Agonist* | 74.5% (38) | 51/51 |
|  | *Istradefylline* | 3.9% (2) | 51/51 |
|  | *Extended-Release Levodopa* | 51% (25) | 51/51 |
| **1 hour of FDMR Dyskinesia** | *Amantadine* | 80% (16) | 20/20 |
|  | *Contraindication to Amantadine* | 20% (4) | 20/20 |

*COMTi – Catechol-O-Methyl Transferase inhibitor, MAO-Bi – Monoamine Oxidase B inhibitors, FD – Functionally Disabling, MR – Medication Refractory, PD – Parkinson’s Disease. For criteria definitions, please see Table 1
